# Hydrothermally-treated soybean-fortified maize-based *nsima* (stiff porridge) could contribute towards alleviating seasonal body weight loss in farming communities in sub-Saharan Africa

**DOI:** 10.1016/j.heliyon.2023.e17737

**Published:** 2023-06-28

**Authors:** Beatrice Mtimuni, Grace Timanyechi Munthali, Aggrey Pemba Gama, Gabriella Chiutsi-Phiri, Numeri Geresomo, Lovemore Nkhata Malunga, Limbikani Matumba

**Affiliations:** a Faculty of Food and Human Sciences, Lilongwe University of Agriculture and Natural Resources (LUANAR), Bunda College, Box 219, Lilongwe, Malawi; b Department of Agricultural Research Services, Chitedze Research Station, P.O. Box 158, Lilongwe, Malawi; c Faculty of Life Sciences and Natural Resources, LUANAR, NRC, Box 143, Lilongwe, Malawi; d Department of Food and Human Nutritional Sciences, University of Manitoba, 209 Human Ecology Building, Winnipeg, MB, R3T 2N2, Canada

**Keywords:** Maize-soy-stiff-porridge, *Nsima*, Fortified, Hydrothermally-treated-soybeans, Farming community, Body-weight

## Abstract

**Objective:**

This study explored the use of hydrothermally-treated soybean-fortified maize-based stiff porridge (nsima) in managing body weight losses among the farming family community in Malawi during the labour-intensive cropping (growing) season. We hypothesized that soybean-fortified maize-based nsima could prevent seasonal body weight losses in farming communities during labour-intensive seasons better than conventional 100% maize nsima.

**Research methods & procedures:**

A single-blind parallel dietary intervention 90-day study. During energy stress months, 42 farming households in Malawi were supplied with 15 kg of blind formulation of soybean-fortified maize flour (soybean: maize, 1:4, wt/wt) per person per month except for under-fives who were allotted half the quantity. Forty households were provided with equivalent quantities of 100% maize flour and served as control. Body weights of participants were taken at baseline and endpoint.

**Results:**

After 3 months, the experimental group registered 3.7, 4.2, 2.9, and 5.2% statistically higher body weight compared to the controls for the under-five, the 5-9-year-olds, the 10-19-year-olds, and the >20-year-olds, respectively.

**Conclusion:**

Soybean-fortified stiff porridge could feasibly be used to alleviate wasting among the resource-constraint populace in Malawi and many other parts of sub-Saharan Africa that rely on maize as a major staple.

## Graphical abstract

**Figure:**
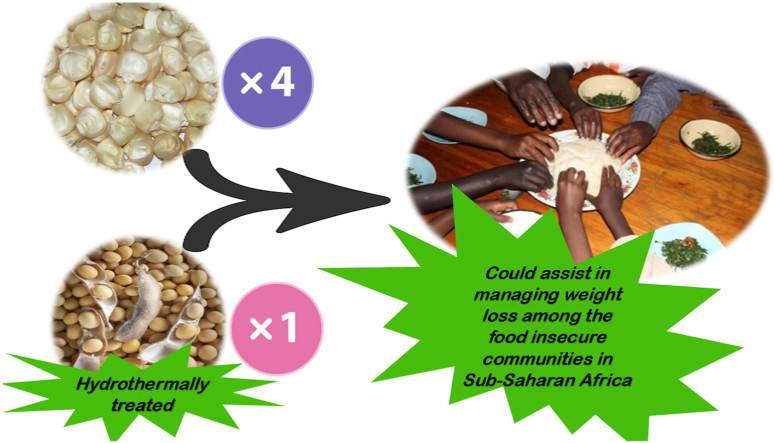

## 1 Introduction

Marked seasonal variations in food security characterize most farming systems in developing countries particularly those that have one dominant crop a year. In these regions, food supply is abundant usually after harvest and scarce during the cropping periods as they wait for the next harvest. Often the food shortages coincide with intense agricultural work leading to a negative energy balance (energy intake insufficient to meet energy expenditure). The negative energy balance results in body wasting in both adults and children and impaired growth in children [[1], [2], [3], [4], [5], [6]]. Seasonal body weight loss in adults and impaired growth in children have significant human costs such as immunosuppression and irreparable damage to cognitive function in children [[7], [8], [9]].

The seasonal loss of body weight of farming communities during the lean periods is exacerbated by over-reliance on cereal staples with few, and sometimes no, foods from the other food groups: vegetables, fruits, legumes and nuts, animal foods, and fats [10,11]. This results in a diet low in the lipids, proteins, vitamins, and minerals needed for an active and healthy life. Legumes, such as soybeans, offer a cheap option for increasing the nutritive value of cereal-based foods. Soybeans contain roughly 40% crude protein which when properly processed have digestibility rates comparable to other high‐quality proteins from such foods as meat, milk, fish, and egg [[12], [13], [14]]. Besides, they contain lipids up to around 20% [15].

Owing to the high nutrient content, soybeans are popularly used to enrich porridge served as breakfast for the whole family and as a complementary food for infants and young children in Sub-Saharan Africa (SSA) [16,17]. However, these porridges principally contain a high volume of water and therefore reduce the net nutrient intake. Unfortunately, traditionally treated soybeans are not acceptable for fortifying *nsima* (also known as *nshima* in Zambia, *sadza* in Zimbabwe, *ugali* in Kenya and Tanzania, *mielie pap* in South Africa), a thick polenta-like dish served at lunch and dinner. Soybeans are usually processed by roasting or extrusion to eliminate anti-nutritional factors. However, these treatments result in tastes and aromas that are unacceptable to consumers in SSA.

Considering this, our research group hydrothermally treated soybeans and optimally incorporated them into maize-based *nsima* successfully. The incorporation of hydrothermally treated soybeans (HS) (up to 20%) was highly accepted by consumers (n = 125) in Malawi [18]. This hydrothermally-treated soybean-fortified maize-based *nsima* (HSMN) was effective in managing HIV-related wasting among resource-poor people [19]. Thus, we hypothesized that HSMN could potentially prevent seasonal body weight losses in farming communities in SSA during labour-intensive seasons better than conventional maize *nsima.* Specifically, the study aimed at assessing a) whether consumption of HSMN during cropping season can alleviate wasting among the resource-constraint farming community, and b) long-term acceptance and the efficacy of the HSMN in managing seasonal body weight losses amongst the broader resource-constrained farming population.

## 2 Methodology

### 2.1 Study area

The study was carried out in two villages located within the Kamwendo Area in the rural district of Mchinji, Central region of the Republic of Malawi. Just like any other place in Malawi, the study area has one rainfall season that stretches from November/December to April/May. The main food crops are maize, groundnuts, and soybeans. Tobacco is the most important non-food crop grown in the area. Cultivation, harvesting, and sales are done by men, women, and children. Land preparation (land clearing and making ridges) is done using hand hoes and is often carried out from September through November. Sowing of maize is carried out in late November or early December. The staple food maize is harvested between May and June. In addition to farming activities, women usually do the housekeeping, take care of the children, prepare food, gather and chop firewood and fetch water while men are mostly engaged in financial management-related activities and socialization.

### 2.2 Study design and area

The single-blind parallel study design was used to study the effect of HSMN on seasonal wasting in farming communities in Malawi. The study was implemented during the energy stress farming season, specifically from 10th September to 11^th^ December 2018. Two villages in the Mchinji District of Malawi were randomly selected from a list of villages under the National Smallholder Farmers' Association of Malawi (NASFAM, the largest smallholder-owned membership organization in Malawi) within Kamwendo, an area renowned for high groundnut production. Mazambani village was assigned as the experimental village while Suwelela Village situated about 15 km away, served as the control village. Two separate villages were used to avoid contamination effects (spill-over effects). The community of the control village had a similar socioeconomic structure to that of the experimental village. The NASFAM system is organized into a unique extension network to support its membership in terms of production, marketing, and community development.

### 2.3 Enrollment of participating households

The study sample size by design was constrained to 40 households each in experimental and control villages due to available financial resources to provide the experimental HSMN and conventional maize flours. Both the experimental village (Masambani) and control village (Suwelela) had greater than 40 households, consequently, the enrollment of households within each village was done sequentially (without jumping a household) from the northern end until a total of 42 and 40 households were reached for experimental and control villages respectively. The nucleation of the food supplementation helped to minimize the sharing of the flours between participating and non-participating households.

### 2.4 Intervention

Participants in the experimental village were given a pack of HSMN flour prepared by mixing four parts of maize and one part of HS. The hydrothermal treatment was performed to eliminate the beany-grassy flavor in the soybeans in the following manner: clean soybeans were gradually introduced into boiling water without causing the water to stop boiling (one-part soybean to three parts water) for an hour, cooled in water, and manually dehulled by scrubbing the soybeans between two hands to force the hulls from the cotyledons. The dehulled soybeans were then washed and sundried for two days and then blended with maize in the ratio of 1:4 (soybean/maize, wt/wt). To ensure the homogeneity of the maize-soybean flour mixtures, the dried hydrothermally processed soybean beans were thoroughly mixed with the maize portion (dehulled maize grits) before the milling using a hammer mill (GM33, Ndume Ltd, Kenya) fitted with a standard sieve (seive #8). The present HSMN formulation was chosen because it was deemed optimal in terms of sensory acceptability [18].

Each participating household in the experimental village was provided with a monthly ration of HSMN flour whose weight was determined by household size and age composition. All participants older than 5 years were assigned 500 g of the HSMN flour per day and younger participants were allotted 250 g/day. Participants in the control cohort were provided equivalent quantities of 100% maize flour monthly rations. The quantity of flour for the >5 years old and under-five groups was based on daily per capita maize consumption of 500 and 250 g/day (dry matter basis), respectively [20]. Participants were instructed to replace their flour with the provided rations, and they were strongly advised not to share it with other households. To ensure compliance with HSMN intake in the experimental group, two interns randomly performed unannounced audits and tasted the HSMN. Similar audits were performed with control households to avoid bias.

Considering that consumer opinions and food acceptability are influenced by how much information has been presented to them about the products [21,22], the participants were not informed that the experimental rations had been fortified with soybeans. However, to screen out soy-allergic consumers without compromising the blinding of participants (regarding the enrichment of maize flour*),* the participants were individually asked to indicate if they have problems with common allergens including fish, pork, meat, egg, peanut, tree nut, wheat, and soy. At the end of the three-month study period, participants in the experimental village were asked to guess what ingredient had been added to the maize flour.

### 2.5 Anthropometry

The weight and height of all participants were measured on initial contact. Bodyweight was measured three times by trained personnel using an electronic scale (SECA GmbH & Co., Hamburg, Germany) while the participant was standing barefoot and in light clothing with an accuracy of 0.1 kg. Similarly, height was measured without shoes and headgear three times while the participant was standing barefoot in an orthostatic position on a stadiometer with an accuracy of 0.1 cm; an average was calculated. For 6–23.9 months olds, the crown-heel length was taken using an infantometer (a flat wooden surface with head and footboards) with subjects in a lying position. Weights and heights for participants aged 19 and below were measured monthly for 3 months. However, for adults, heights were measured at baseline only while weight measurements were taken at the end of each of the three months since their heights were not expected to change.

Weight-for-age z-scores (WAZ), height-for-age z-scores (HAZ), BAZ and weight-for-height z-scores (WHZ) were calculated using the software WHO Anthro version 3.1.0 and WHO AnthroPlus version 1.0.3 (World Health Organisation, Geneva, Switzerland. For adults, BMI was calculated using the standard formula: BMI = (weight (kg)/height^2^ (m^2^)) and interpreted according to the WHO classification. Underweight (prevalence of thinness) was classified as BMI <18.5 and severe underweight as BMI <16. Overweight was classified as BMI of 25.0–29.9 and obesity as BMI ≥30.0. For adolescents aged 10–19 years, their weights, heights, and ages were used to generate BMI-for-age Z-scores using the WHO anthropometric calculator [23]. Underweight was classified as BMI-for-age less than −2 SD and severe underweight as BMI-for-age less than −3 SD. Overweight was classified as BMI-for-age > +2 SD and obesity as BMI-for-age > + 3 SD.

### 2.6 Ethical consideration

The study design was approved by the National Health Science Research Committee under the Ministry of Health and Population and is registered with the US Office for Human Research Protections (OHRP) as international IRB number IRB00003905FWA00005976 and study number NHSRC#18/01/1951. The study design also complies with the 1964 Declaration of Helsinki and its later amendments or comparable ethical standards. Permission to do the study was also provided by the Mchinji District Commissioner and local authorities. Written informed consent was sought from the household head for households’ participation after a detailed explanation of the purpose of the study. No subjects declined to give consent to participate in the study.

### 2.7 Data analysis

Non-parametric tests were used in this study as the data did not satisfy the normality assumption, Kruskal-Wallis test was used to determine the effect of the treatment on cumulative percentage weight gain over the three-month period. When significant main effects were present, this was followed by Dunn's post hoc test for multiple comparison. Further, the overall changes in various nutritional status indicators (WHZ, WAZ, HAZ, BAZ, and BMI) between the experimental and control villages was determined using Mann-Whitney *U* test. Hypotheses were tested at 5% (0.05) significance level. All statistical analyses were performed with SPSS version 23 (IBM Corp, Armonk, New York).

## 5 Results

**Fig. 1:**
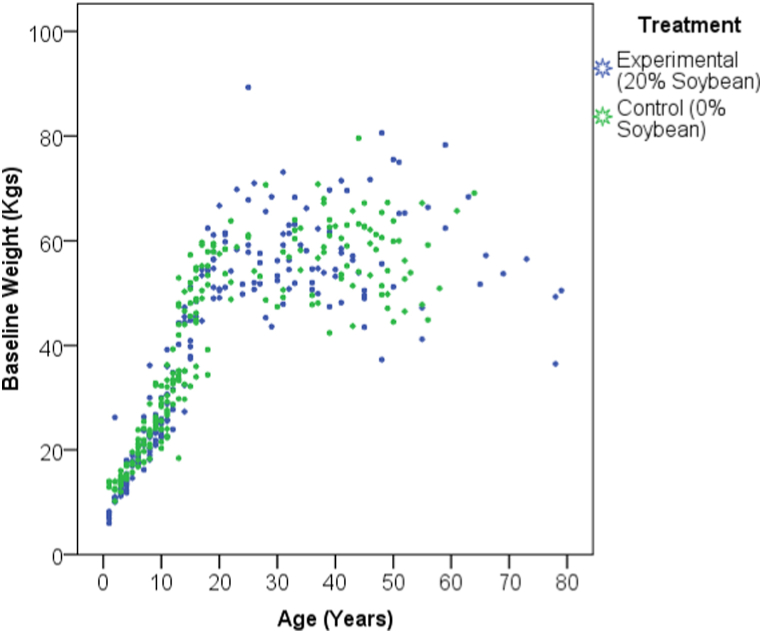
Scatter plot of control and experimental populations showing differential increase in weight with age at baseline.

A total of 42 households in the experimental village and 40 households in the control village were approached and recruited into the study. This translated to a total of 192 and 208 participants in the experimental and control villages, respectively. As shown in the scatter plots, most of the adults in both the experimental and control groups had baseline body weights of less than 60 kg (Fig. 1). Participants in the experimental group consistently consumed the HSMN for the entire period as verified by random unannounced monitoring visits performed by resident interns who visited at least each household twice during mealtimes. Most importantly, no participant recognized that soybeans were incorporated into the maize-based flour provided to them.

Weight by age categories for the under 20-year-olds (under 10-year-olds based on weight-for-age z-score, and adolescents aged 10–19 year olds based on BMI for age); and the adults 20 years and older (based on BMI) at baseline are graphically presented in Table 1. Over 85% of the participants in each of the three age categories below 20 years of age (under 5 years; 5–9 years and 10–19 years) in both the experimental and control group had normal weight. Similarly, most of the adults had normal body weights at baseline. However, the mean BMI for male participants (21.9 ± 2.4) was significantly (*p*<*0.01*) lower compared to that of females (23.2 ± 3.4). In fact, 24.7% of the females in this age category (≥20 years) were either overweight or obese. Comparatively, fewer males were overweight (9.5%) and none was obese.

**Table 1:** Nutritional status of participants at baseline.

Age category	Nutrition status	Gender	Number of individuals	
			0% Soybean	20% Soybean
6 months - <5years (WHZ)	Severe wasting	Female	0	1
	Normal weight	Male	9	11
		Female	12	14
	Overweight	Male	1	0
5–9 years (BAZ)	Normal weight	Male	20	25
		Female	19	10
10–19 years (BAZ)	Moderate underweight	Male	1	1
		Female	2	0
	Normal weight	Male	37	22
		Female	24	18
Adults (BMI)	Severe underweight	Male	0	1
		Female	0	2
	Moderate underweight	Male	3	2
		Female	0	3
	Normal weight	Male	35	35
		Female	30	32
	Overweight	Male	3	5
		Female	11	7
	Moderate obese	Female	1	2
	Obese	Female	0	1

Subtle divergent shifts in weight between the experimental and the control group were observed over the 3-months study period. While the experimental population subtly shifted toward overweight, the controls slightly shifted towards underweight (Table 2). These BMI category shifts were well collaborated by cumulative monthly mean weight gains or losses (percentage) the populations experienced (Table 3). The under 20 years old control cumulatively gained weight at significantly lower rates than the experimental groups (Table 3). The adults within the control group cumulatively lost weight (Table 3). However, the experimental group cumulatively gained weight during the study period. Importantly, the mean weight differences between the experimental and control groups at the end of three months were statistically significant.

**Table 2:** Change in nutritional status indicators for participants over a 3-month study period.

Age group	Nutritional status Indicator	Treatment								Treatment Effect on change using Mann-Whitney Test	
		0% Soybean				20% Soybean					
		*N*	Baseline Mean ± SD	Mean Change	Mean Change Range	*n*	Baseline Mean ± SD	Mean Change	Mean Change Range	U	*p*-Value
Under 5 yrs.	WHZ	22	0.705 ± 0.724	−0.088	−0.850 – 0.710	26	0.320 ± 0.742	0.360	−0.760 – 1.410	412	0.009
	WAZ	22	−0.285 ± 0.777	0.122	−0.620 – 2.480	26	−0.375 ± 0.960	0.556	−0.150 – 3.960	469	0.000
	HAZ	22	−1.504 ± 0.874	0.226	−0.360 – 2.430	26	−1.049 ± 2.100	0.543	−0.860 – 0.710	433	0.002
	BAZ	22	0.841 ± 0.786	−0.108	−0.110 – 0.750	26	0.471 ± 0.670	0.345	−1.03 – 1.790	409	0.011
5–9 yrs.	WAZ	39	−0.949 ± 0.970	0.097	−0.63 – 2.140	35	−1.101 ± 0.964	0.430	−0.130 – 2.040	1125	<0.0001
	HAZ	39	−1.533 ± 1.076	−0.004	−0.230 – 0.380	35	−1.418 ± 1.056	0.163	−0.400 – 1.690	1122	<0.0001
	BAZ	39	0.034 ± 0.763	0.073	−0.970 – 2.230	35	−0.281 ± 1.015	0.430	−0.630 – 1.210	1019.5	0.000
10–19 yrs.	BAZ-Male	38	−0.573 ± 0.856	0.070	−0.940 – 0.620	23	−0.705 ± 0.544	0.420	−0.380 – 1.130	674.5	<0.0001
	BAZ-Female	26	−0.750 ± 1.137	0.170	−0.220 – 0.540	18	−0.302 ± 0.903	0.315	−0.140 – 0.780	546.5	0.004
20 yrs and above	BMI-Male	38	21.839 ± 2.169	−0.314	−2844 – 1.187	43	21.959 ± 2.599	0.874	0.202–2.876	1665.0	<0.0001
	BMI-Female	42	23.439 ± 2.973	−0.339	−2.844 – 1.187	46	23.021 ± 3.835	0.797	0.132–2.292	1764.0	<0.0001

**Table 3:** Cumulative monthly mean weight gains (percentage) for participants over a 3-month study period.

Age Group	Gender	Month	Number of participants		Cumulative monthly mean weight gain/loss (%)	
			20% Soybean	0% Soybean	20% Soybean	0% Soybean
Under 5 years	All	1st Month	26	22	4.4^b^	−0.2^b^
		2nd Month	26	22	8.2^a^	0^b^
		3rd Month	26	22	10.7^a^	5.9^a^
5–9 years	All	1st Month	35	39	2.5^c^	0.3^b^
		2nd Month	35	39	4.5^b^	0.7^b^
		3rd Month	35	39	8.1^a^	4.1^a^
10–19 years	Male	1st Month	23	38	2.5^b^	0^b^
		2nd Month	23	38	3.9^b^	−0.1^b^
		3rd Month	23	38	6.2^a^	1.8^a^
	Female	1st Month	18	26	1.0^c^	−0.1^b^
		2nd Month	18	26	2.7^b^	0.4^b^
		3rd Month	18	26	5.1^a^	3.0^a^
20 years and above	Male	1st Month	43	38	1.1^c^	−0.5^a^
		2nd Month	43	38	2.9^b^	−1.5^a^
		3rd Month	43	38	4.1^a^	−1.4^a^
	Female	1st Month	46	42	1.0^c^	−0.5^a^
		2nd Month	46	42	2.1^b^	−1.6^a^
		3rd Month	46	42	3.4^a^	−1.5^a^

Note: Means with different superscripts (a, b and c) are significantly different (p < 0.05) within each sub-column using Kruskal-Wallis’ test (mean ranks) followed by Dunn's post Hoc test.

## 6 Discussion

While affluent, technically advanced societies have escaped from the effects of nutritional seasonality, farming communities in most developing countries continue to struggle with under-nutrition during cropping seasons. As such, a search for effective interventions continues to be a high priority. The primary objective of this study was to examine the feasibility of utilizing HSMN in managing seasonal body weight losses amongst the farming population in SSA. This study was active from September to December, a period when the participants were involved in energy-intensive land preparation using hand hoes. While children (mainly adolescents) from participating households were involved in the land preparation, most of the work was performed by adults as children were partly preoccupied with school.

Seasonal bodyweight loss is often associated with insufficient food supply, which results in negative energy balance. The continuous weight loss observed in the adults and faltered growth in children of the control group suggests that the reliance on ordinary *nsima* as the main staple by the farming communities failed to meet their energy demands during the labour intensive period. It was anticipated that the energy difference would be provided by other food eaten together with *nsima* or independently. However, the impaired growth and loss of weight observed in the control group during the study period suggest that they were reliant on *nsima* as a principal source of energy. This was more pronounced in children as their growth faltered during the study period. Thus, our study suggests that seasonal body weight loss and impaired growth rate observed during cropping season cannot be addressed by supplying enough staple food (*i.e.* maize in this case) alone.

One option to address seasonal weight loss while satisfying the culture's dependency on maize *nsima*, is to alter the recipe so that the *nsima* provides more protein, fat, vitamins, and minerals.

Therefore, we investigated the possibility of substituting 20% of maize with HS to increase *nsima's* energy and nutrition density. The resultant HSMN was well accepted by the community such that they were able to consume it twice a day throughout the study period. Furthermore, the results of this study suggest that HSMN was effective in alleviating seasonal body weight loss in adults in our experimental group. Also, the HSMN successfully supported the children's growth as demonstrated by continued weight gain by children during the study period. The HSMN not only contains higher amounts of protein but also higher content of lipids and micronutrients than the common maize-based *nsima*. Since soybean contains approximately 40% proteins and 20% lipids [15], while maize contains only about 10% proteins and 4.5% lipids [24], the 20% soybean *nsima* typically represents a 60% boost in protein and 69% in lipid composition (flour blend compositions: 16.0% protein and 7.6% lipid).

During peak cropping seasons, adults in Malawi and many parts of SSA may work in the field 8–12 h per day; but after harvest, they do not go to the fields. Ironically, food is plentiful after the harvest, but with storage losses, food wastage, and use, little is left during the peak cropping seasons. Therefore, greater body weight loss than those observed in the present control cohorts is more likely to occur since a significant proportion of farmers may have even lacked the plain maize supplied to the controls. Given that the BMIs of a significant proportion of farmers in SSA are already skewed toward the bottom of the normal range, the risk of the BMI falling below 17 is likely. The BMI below 17 constitutes a health risk and may lead to impaired physical capacity [25,26]. It is worth highlighting that the actual body weight loss suffered by a typical farmer in the area was not elucidated since the study design omitted the ‘usual care’ group (not given any additional food rations from the researchers). Nevertheless, much greater seasonal weight losses have been documented in other parts of SSA [ [4,[27], [28], [29]]].

It is worth highlighting here that the current study did not capture household dietary diversity score (HDDS) and individual dietary diversity score (IDDS) for the population. These data could have reflected household food accessibility and diet quality. While such data could have provided a picture of what and how much the population was consuming, and how much the *nsima* contributed toward the total calories, it was beyond the study's scope. This study focused on comparing the efficacy of HSMN against conventional plain maize–based *nsima* in managing weight loss during a labor-intensive farming period using a population from the same socio-demographic structure. The finding of a low prevalence of undernutrition among the current study population does not reflect the general status of Malawians. While only 1 out of 48 under five years olds was found to be undernourished, typically in Malawi, 37% of children under the age of five years are stunted [30]. This is likely to have been because the study population consisted of fairly progressive farmers belonging to NASFAM (farmer association), whose agricultural productivity is comparatively better than that of the general population due to better access to advisory services. However, the choice of the study population was the most ideal since this study was primarily aimed at evaluating the efficacy of the HSMN in managing seasonal body weight losses arising from intensive physical agricultural workloads.

The failure by participants to recognize that soybeans were incorporated into the HSMN, coupled with the fact that the participants continued to consume the HSMN for the entire study period (3 months), is a clear demonstration that the soybean deodorization process employed was efficient and did not compromise sensory properties of the HSMN. Unlike roasting, the hydrothermal treatment of soybeans did not result in the formation of other extraneous flavors that may arise from browning reactions such as the Maillard reaction. Considering that soybeans are produced almost everywhere across SSA, and the simplicity of the soybean hydrothermal treatment, promoting the HSMN amongst SSA's agrarian communities seems plausible.

## 7 Conclusion

The current results support the hypothesis that HSMN could contribute towards alleviating wasting among food insecure agrarian communities during labour-intensive seasons than conventional plain maize–based *nsima.* The weights of the participants who consumed HSMN rose steadily over the study period and varied with age group. However, different age groups responded differently in terms of BMI after consuming HSMN during the study. The amount of HS to be incorporated into *nsima* should be based on the nutrition assessment of the specific groups to maintain a healthy BMI. Therefore, it will be important to conduct comprehensive randomized controlled trials involving broader populations, including those living in urban areas, to better understand the effect of the HSMN on the nutritional status of various gender categories involved in different occupations. Further, it is imperative to perform economic and adoption barrier analyses and shelf-life studies, before the intervention is made available broadly.

## Funding statement

The authors gratefully acknowledge the funding support to carry out this study from Programme for Accompanying Research in Innovations (PARI) project. PARI Project is coordinated by The Forum for Agricultural Research in Africa (FARA) in Africa and Center for Development Research (ZEF).

## Author contribution statement

Beatrice Mtimuni, Grace Timanyechi Munthali, Limbikani Matumba: Conceived and designed the experiments; Performed the experiments; Analyzed and interpreted the data; Contributed reagents, materials, analysis tools or data; Wrote the paper. Aggrey Pemba Gama, Lovemore Nkhata Malunga: Conceived and designed the experiments; Performed the experiments; Analyzed and interpreted the data; Wrote the paper. Gabriella Chiutsi-Phiri, Numeri Geresomo: Conceived and designed the experiments; Performed the experiments; Wrote the paper.

## Data availability statement

Data will be made available on request.

## Declaration of competing interest

The authors declare that they have no known competing financial interests or personal relationships that could have appeared to influence the work reported in this paper.
